# Diabetic Ketoacidosis in an Euglycemic Patient

**DOI:** 10.7759/cureus.10065

**Published:** 2020-08-27

**Authors:** Hassan Mumtaz, Muhammad Ahsan Shafiq, Hajra Batool, Tayyaba Naz, Saima Ambreen

**Affiliations:** 1 Internal Medicine, Rawalpindi Medical University, Rawalpindi, PAK; 2 Internal Medicine, Rawalpindi Medical University, Islamabad, PAK; 3 Medicine, Rawalpindi Medical University, Rawalpindi, PAK

**Keywords:** edka, dka

## Abstract

Diabetes is a common disease, and the number of patients is increasing every year. We report a case of a 34-year-old man with a history of diabetes mellitus (diagnosed at eight years old) and was on treatment with tablet glimepride with poor compliance. The patient sought consultation due to vomiting and abdominal pain 12 hours after onset along with burning micturition for four days. His initial blood sugar random (BSR) level was 84 mg/dL. Further lab tests revealed pH: 7.14, bicarbonate: 6.4 mEq/dL, sodium: 141, potasium: 3.8, chloride: 107, PO2: 115, PCO2: 19.4, serum amylase: 51, base excess (BE): -21.3 mmol/L, and positive ketonemia, i.e. 1.39. He was reanimated with parenteral crystalloids and insulin infusion. Eventually with subsequent arterial blood gases (ABGs) and ketones, the patient got better and was eventually declared to be out of diabetic ketoacidosis (DKA) and later discharged. There are very less studies done on euglycemic DKA (eu-DKA); so many physicians fail to diagnose the patients properly and they fall into the invisible cases chunk.

## Introduction

Diabetic ketoacidosis (DKA) is a well-known complication in diabetics. Its diagnosis requires two things: arterial blood gases (ABGs) that shows increased anion gap metabolic acidosis and presence of ketone bodies in blood or urine. Typically it presents with hyperglycemia. The clinical presentation of this pathology is diverse, going from abdominal pain to sensory deterioration and coma [1]. Atypically, in some rare instances, it can present with patient being euglycemic but having ketoacidosis. This pathology was first described by Munro in 1973 [2] but, in his work, he studied patients with blood glucose levels under 300 mg/dL. Currently, it is defined with blood glucose levels under 250 mg/dL. Some 6% of patients show blood glucose levels under 300 mg/dL and around 1% of patients show levels under 180 mg/dL. The most common causes are insulin administration on the way to the hospital and fasting [1]. This pathology was first described by Munro having patients’ blood glucose levels under 300 mg/dL in his study [3]. In 1973, Munro et al. proposed a new type of DKA that is not accompanied by hyperglycemia called euglycemic DKA (eu-DKA) [3]. eu-DKA is characterized by a mild increase in blood glucose; thus, DKA cannot be suspected based on the markedly elevated glucose, which could result in delayed diagnosis and a delay in starting the treatment [4].

In his study 6% of patients showed blood glucose levels under 300 mg/dL and around 1% of patients showed levels under 180 mg/dL. The most common cause was insulin administration on the way to the hospital and fasting [2].

## Case presentation

A 34-year-old man who presented in Medical Emergency of Holy Family Hospital, affiliated with Rawalpindi Medical University, Punjab, Pakistan with a history of diabetes mellitus (diagnosed at eight years old) was on treatment with tablet glimepride with poor compliance. 

The patient sought consultation due to vomiting and abdominal pain 12 hours after onset along with burning micturition for four days. Upon physical examination, the abdomen was soft with diffuse pain and no signs of peritoneal irritation. Fever, cough, and loose motions were ruled out. Laboratory results showed the following values: Hb: 15.2, WBC: 11.3, platelets 144, blood glucose: 84 mg/dL, total bilirubin: 0.8, urea: 23, pH: 7.14; bicarbonate: 6.4 mEq/dL, sodium: 141, potasium: 3.8, chloride: 107, PO2: 115, PCO2: 19.4, serum amylase: 51, base excess (BE): -21.3 mmol/L, and positive ketonemia, i.e. 1.39.

Upon diagnosis of normoglycemic diabetic ketoacidosis, reanimation was started according to the American Diabetes Society Guidelines for DKA 2009 with parenteral crystalloids administered at 250 mL/h during 24 h. It was interspersed with isotonic saline solutions and polyelectrolyte solutions. Insulin infusion was also given at 0.14/kg/h. Total income was 7000 mL/24 h. Urinary volume was 2750 mL/24 h. Positive balance was 4250 mL/24 h. Progress was shown with improvement of the clinical condition and lab monitoring every eight hours: pH 7.16; bicarbonate of 4.9 mmol/L, PCO2: 15.3 mmHg, PO2: 118 mmHg, and BE: -22.5 mmol/L with blood glucose levels in the normal range 172 mg/dL (< 200 mg/dL). Eventually with subsequent ABGs and ketones, the patient got better and was eventually declared to be out of DKA and later discharged (Table 1).

**Table 1:** Before and after ABGs. ABGs, arterial blood gases; BSR, blood sugar random; BE, base excess

	pH	HCO3	PO2	PCO2	BSR	BE	Ketonemia
At admission time	7.14	6.4	115	19.4	84	-21.3	+ive
At discharge	7.39	25	94	36	172	+2	-ive

Triggering factor 

Inviting event would be urinary tract infection (UTI) because the patient complained of burning micturition for four days.

## Discussion

Euglycemic diabetic ketoacidosis is a clinical triad comprising increased anion gap metabolic acidosis, ketonemia or ketonuria, and normal blood glucose levels <200 mg/dL. This condition is a diagnostic challenge as euglycemia masquerades the underlying DKA [5]. The exact mechanism of eu-DKA is not entirely known, but has been associated with partial treatment of diabetes, carbohydrate food restriction, alcohol intake, and inhibition of gluconeogenesis. eu-DKA can also be associated with sodium-glucose cotransporter 2 (SGLT-2) inhibitor medications and sulfonylurea medications. 

Euglycemic diabetic ketoacidosis is a diagnostic challenge for treating physicians, as there is no hyperglycemia. On the other hand, there are many causes of metabolic acidosis in patients in the ICU, although, when analyzing the gap, high gap metabolic acidosis is less frequent than hyperchloremic acidosis [6]. Therefore, knowing this pathology is key when treating patients with diabetes. Moreover, the triggers are varied (Table 2).

**Table 2:** Causes of eu-DKA [7]. eu-DKA, euglycemic diabetec ketoacidosis; SGLT-2, sodium-glucose cotransporter 2

Fasting
Insulin use prior to hospital admission
Pregnancy
Use of SGLT-2
Cocaine abuse
Pancreatitis
Cirrhosis
Use of insulin pump
Sepsis

Approximately 2.6%-3.2% of DKA admissions are euglycemic [8-9]. DKA-associated with SGLT2 inhibitors has rates ranging from 0.16 to 0.76 events per 1000 patient-years in patients with type 2 diabetes. Blau et al. estimate that the SGLT2 inhibitors increase the risk of DKA in type 2 diabetes patients seven-fold [10].

Another possible cause of eu-DKA is the administration of insulin before being admitted to the hospital [11]. Other causes are pancreatic lesions developed during pancreatitis due to alcohol consumption, associated with the fasting required by this condition, which would explain the development of eu-DKA [11]. Furthermore, cocaine abuse causes an increase in the secretion of cortisol and noradrenaline by the adrenal gland, in addition to the anorexigenic effects of this drug, which lead to fasting [12].

In a study based in New Jersey, United States, the initial fluid replacement followed was continuous IV insulin infusion at a rate of 0.02-0.05 units/kg/h [4]. Dextrose-containing fluids was started along with the insulin infusion to avoid hypoglycemia, with a target serum glucose level of 150-200 mg/dL. Resolution of eu-DKA was identified by the presence of two of the following: a serum bicarbonate level ≥ 15 mmol/L, an anion gap ≤ 12 mmol/L, or a venous pH > 7.3 [13-14].

We treated the patient with parenteral crystalloids administered at 250 mL/h during 24 h. It was interspersed isotonic saline solutions and polyelectrolyte solutions, along with insulin infusion given at 0.14/kg/h.

Many cases are not treated properly because no database is available for it.

## Conclusions

Euglycemic diabetic ketoacidosis is a diagnostic challenge, not only due to the absence of its most important sign, which is hyperglycemia, but also due to its varied triggers. Knowing the different contexts in which it can occur will allow us to suspect eu-DKA and begin rapid and adequate treatment of the precipitating cause, as well as aggressive hydration, glucose homeostasis through insulin administration, and adjustment of electrolyte imbalances. 

Morbidity and mortality can be significantly improved by early diagnosis and initiation of treatment. Sepsis, toxic alcohol ingestion, and alcoholic ketoacidosis must be ruled out in order to take great care in patients presenting with a euglycemic anion gap acidosis. Early IV crystalloid and prompt initiation of insulin and dextrose infusion are the primary treatment. There are very less studies on eu-DKA so many patients fall into the invisible cases chunk. More studies should be done on eu-DKA so that there are no patients left undiagnosed.
